# Sequencing of historical plastid genomes reveal exceptional genetic diversity in early domesticated rye plants

**DOI:** 10.1016/j.isci.2025.112716

**Published:** 2025-06-06

**Authors:** Jovan Komluski, Sofia Filatova, Frank Schlütz, Benjamin Claaßen, Manfred Rösch, Ben Krause-Kyora, Wiebke Kirleis, Eva H. Stukenbrock

**Affiliations:** 1 Botanical Institute, Kiel University, 241148 Kiel, Germany; 2 Max Planck Institute for Evolutionary Biology, 24306 Plön, Germany; 3 Groningen Institute of Archaeology, Groningen, the Netherlands; 4 Institute for Prehistoric and Protohistoric Archaeology, Kiel University, 24118 Kiel, Germany; 5 Archaeobotanical Laboratory of the State Office for Cultural Heritage Baden Württemberg, 78343 Hemmenhofen/Gaienhofen, Germany; 6 Institute of Clinical Molecular Biology, Kiel University, 24118 Kiel, Germany

**Keywords:** Plant genetics, Plant biogeography, Plant evolution

## Abstract

In medieval central Europe, rye was one of the most important agricultural crops. We combined archaeobotanical methods and ancient DNA sequencing of historical rye material to study patterns of genetic diversity across four centuries. We applied archaeobotanical methods to characterize rye material acquired from construction material ranging from the 14th to 18th centuries from different locations in Germany. Next, we extracted DNA to sequence complete chloroplast genomes of six individual samples and compared sequences of historical rye samples to chloroplast genomes of other cereal crops, including a modern rye cultivar. Comparing the aDNA chloroplast samples with modern and non-domesticated rye chloroplast, we show that genetic variation in the historical German rye population was considerably higher. The exceptional difference in levels of genetic variation likely reflects the consequences of late domestication and selective breeding on genetic variation in this important crop in the last few centuries.

## Highlights

• Historical rye samples were obtained from half-timbered houses
• Archaeobotanical and aDNA analyses provided insights into their genetic diversity
• Sequence analyses reveal expectational diversity in historical rye samples
• Domestication has reduced genetic variation in rye within few centuries

## Graphical abstract

**Figure:**
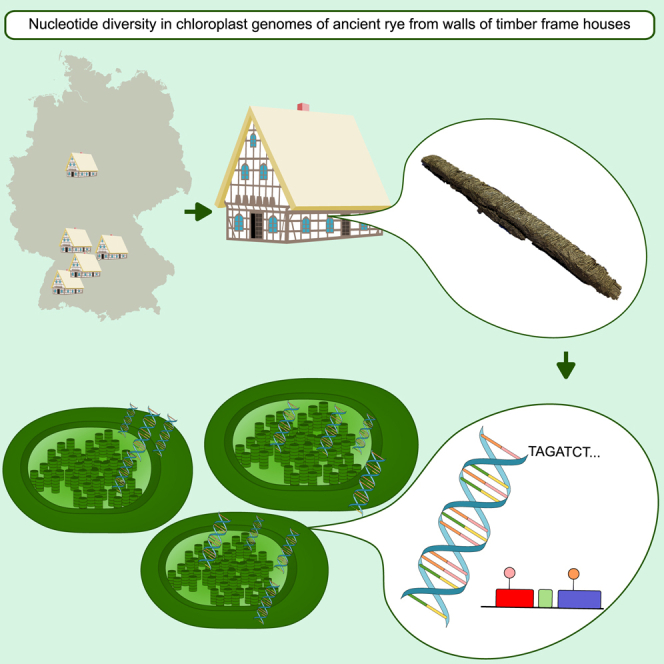

## Introduction

Rye, *Secale cereale,* is an important crop in eastern, central, and northern Europe. It is produced in other regions including North and South America and China, although to a far lesser extent than in Europe. Rye is mainly cultivated for bread production, for beer brewing and whiskey distillation, as well as for animal fodder. The rye plant thrives in nutrient poor soils, can resist frost, and is known to be resistant to many fungal diseases making it a valuable agricultural crop.^1^^,^^2^

The evolutionary history of rye has been much disputed, but ample evidence suggests that rye was domesticated from weedy rye species rather from wild species.^3^ Wild *S. cereale* relatives occur in the “Fertile Crescent” region along with wild relatives of wheat and barley. This indicates that the three cereal species share the same center of origin. However, archeological remains of rye, from the pre-pottery Neolithic, are always found along with remains of either barley or wheat.^3^^,^^4^

*Secale cereale* ssp. *vavilovii* is considered the ancestor of domesticated rye, occurring in parts of Armenia and Eastern Turkey.^5^^,^^6^^,^^7^ The domesticated form of rye first appeared during the seventh millennium BC as an admixture in stores of domesticated einkorn (*Triticum monococcum* ssp. *monococcum*) and emmer (*Triticum turgidum* ssp. *dicoccum*) from South Central Anatolia and the Upper Euphrates valley in Syria,^8^^,^^9^ suggesting that it developed traits of domestication while growing as an arable weed.^8^ Intriguingly, recent studies have indicated the gathering or cultivation of a perennial species of wild rye (*Secale strictum*) as early as the Natufian (ca. 11,100–10,000 BC) in the Middle Euphrates area.^10^ The earliest evidence of cultivation targeted at domesticated rye dates back to the fourth and second millennium BC in Central Anatolia, but based on current archaeobotanical evidence it was most likely a sporadic practice that did not yet establish itself as firmly as the cultivation of barley and species of wheat.^7^^,^^11^

After having entered Europe as an arable weed around the fifth millennium BC and reaching areas north of the Alps, rye retained its status as an arable weed for the next thousands of years.^12^ Occasional archaeobotanical finds of rye grains as weedy admixtures have been found to increase in numbers from 2000 BC to AD 400, within the last two millennia, gradually developing into one of the main staple crops in medieval Europe.^12^^,^^13^ The turn of rye from a weed into a domesticated crop in the course of the Iron Age may have been favored by colder weather conditions, a permanent field culture, and a change in harvest practices with the introduction of iron tools. Since it was not domesticated directly from its wild ancestors, like barley and wheat, rye is attributed a “secondary domesticate”, i.e., a plant with domestication traits that is initially considered an arable weed but eventually develops into an intentionally sown crop in various regions independently, outside of its area of origin.^14^^,^^15^^,^^16^ First archaeobotanical assemblages dominated by rye are known from northern-central Europe from about the second century AD onward and prove the development and use of rye as a crop.^12^^,^^17^^,^^18^

In the 12th–13th century, rye was one of the major crops grown in Europe, likely favored by its “robustness” and tolerance to unfavorable environmental conditions. As documented by pollen records, rye became the main cereal throughout most of northern and central Germany around AD 1000 and it was not until the middle of the 20th century that rye was replaced by bread wheat in its dominant position.^19^ Due to cross-pollination and the transportation of pollen by the wind over long distances,^20^ it took until the middle of the 19th century for first successful targeted selection of varieties from landraces like the Probstei rye. Around 1880, intensified breeding efforts led to rye varieties with greater resistance to lodging,^2^^,^^21^ and a 100 years later, around 1980, hybrid breeding resulted in a considerable increase in yield, but also in greater genetic uniformity, especially uniformity of the cytoplasm.^2^^,^^19^ Today, some wild rye taxa are used as gene sources for crop improvement by interspecific and intergeneric crosses with barley and wheat.^2^

Genetic analyses of rye have been greatly facilitated by the availability of a reference genome sequence.^22^ The exceptionally large genome of rye, approximately 7.8 Gb, has been used to map genetic variants of different rye cultivars, land races, and wild sister species.^23^ By including whole genome data from the weedy rye species *S. cereale* subsp. *vavilovii* and from the wild rye species, *S. strictum* and *S. sylvestris*, Li et al. demonstrated that domesticated rye mostly likely derived from the weed *S. cereale* subsp. *vavilovii* supporting previous conclusions that were derived from analyses of archeological remains.^22^

In the medieval Europe, rye was harvested for the grains, but the up to 2-meter-tall cereal crop has been used for other purposes.^12^ Half-timbered houses have been built in Europe for centuries, and some well-preserved buildings dating back to medieval times can still be found. Infill material of these constructions generally comprised a mixture of clay and chalk with a binder such as grass or straw and water. Straws of rye have been popular as infill material in half-timbered houses, and can, today, be found inside well-preserved constructions. Such remains of rye and other straw material in half-timbered houses from medieval times provide a unique opportunity to investigate the diversity of rye as it was centuries ago. In this study, we explored the access to so-called “*Wellerhölzer*” from half-timbered houses collected at different sites in Germany and representing different epochs ranging from the 14th to the 19th centuries.

In this study, we made use of ancient DNA sequencing approaches to reconstruct chloroplast genomes from different rye individuals and we combined comparative genetic analyses with archaeobotanical descriptions of the well-preserved plant remains. We demonstrate extensive genetic variation in the historical rye chloroplast DNA, including variable sites in protein coding genes. Moreover, we show that the composition of plant remains suggest that rye was sown in highly disturbed fields and likely cultivated as a winter cereal. Our study points to the great potential of ancient DNA analyses and archaeobotanical investigations to unravel the composition and history of agricultural crops.

## Results

**Figure 1:**
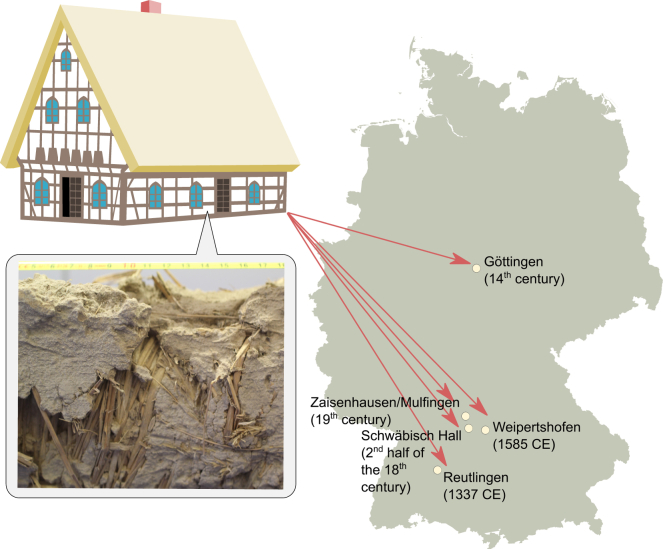
Sampling sites and ages of *Wellerhölzer* plant material used in the study Historical rye varieties were collected from construction material of traditional half-timbered German houses from five locations designated on the map.

The results presented here encompass archaeobotanical and molecular analysis of plant remains from half-timbered houses (*Wellerhölzer*) from five locations in central and south Germany dating from the 14th to 19th centuries AD (Figure 1). Previous archaeobotanical analyses were conducted on thousands of remains from *Wellerhölzer material*, and were combined here with molecular analyses focusing on the chloroplast sequences of plant remains identified as rye, wheat, and barley.

### Archaeobotanical analyses reveal patterns of crop rotation

The individual archaeobotanical analyses have shed light on the conditions in which the rye grew, including soil pH, the season of cultivation and, for a part, the likely cultivation methods. Based on ecological characteristics of the arable weeds accompanying the rye, it was defined that rye was cultivated on moderately basic soils in Zaisenhausen/Mulfingen^24^ and Göttingen,^25^ while it grew on more acidic soils in Weipertshofen,^26^ Reutlingen,^27^ and Schwäbisch Hall (Appendix 1; Table S2).^28^ In all cases, the seasonality of the arable weeds indicates that the rye was sown in autumn as a winter cereal. The crop spectra uncovered from the investigated construction components indicate that it is likely that winter rye was grown in rotation with summer crops such as oat (*Avena sativa*) and barley (*Hordeum vulgare*), possibly in a three-field system. In Göttingen (samples from the 14^th^ century), the analysis furthermore indicated that the soil was likely manured and worked with a moldboard plow. Taken together, archaeobotanical analyses suggest that, aside from variation in soil pH, rye was cultivated using similar methods and in the same season across all the investigated locations and historical times in Germany. The variation in soil pH can be explained by the type of soils available for crop cultivation in the respective regions as well as by the addition of manure.^25^^,^^27^

### Exceptional variation in *Wellerhölzer* rye chloroplast genomes

**Figure 2:**
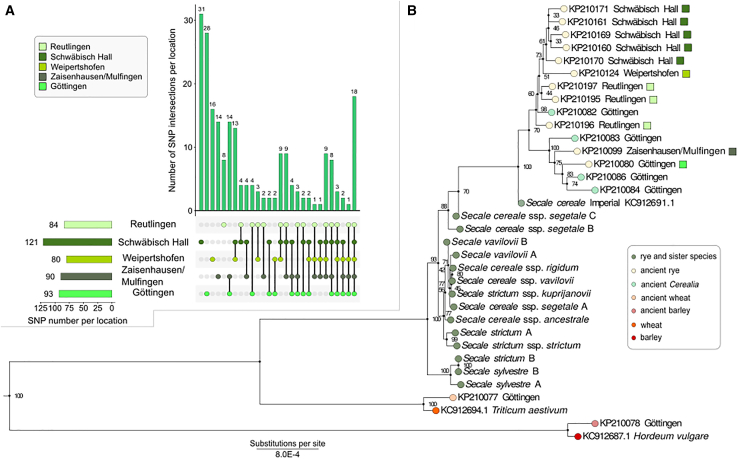
Chloroplast sequence variability in the ancient *Wellerhölzer* rye (A) Upset plot showing intersections between ancient SNPs in the *Wellerhölzer* rye samples grouped based on their sampling location. (B) Neighbor-Joining tree displaying the genetic relationship between the chloroplast sequences of the historical *Wellerhölzer* cereals, rye cv. Imperial and rye relative species. Branch node colors designate the chloroplast sequences of the respective cereal species in accord to the legend. The numbers above branches represent the branch support value.

To assess the overall genetic variation among the eleven *Wellerhölzer* rye samples, we first identified single nucleotide polymorphisms (SNPs) in the chloroplast genomes and grouped SNP intersections based on the sampling site (Figure 2A). 45% of the identified SNPs (97 out of 217) were unique to the sampling site (i.e., variants at certain chloroplast genome positions occurred only in samples from a specific location). Hereby, we find that the highest total number of SNPs as well as highest number of unique SNPs occurs in samples from Schwäbisch Hall (121 SNPs in total of which 31 SNPs are unique to the site) dating back to ca CE 1750. The higher number of variants from this site may be explained with the larger number of samples (five samples). The most distinct samples came from other sites. These were the KP210080 samples from Göttingen and the KP210099 and KP210124 samples from Zaisenhausen/Mulfingen and Weipertshofen, which represented 93, 90, and 80 SNPs, respectively, relative to the rye chloroplast reference genome (Figure S1). The KP210080 sample from Göttingen was collected from the town hall that dates back to the 14th century and it is plausible to speculate that exactly this sample is more distinct because it is older (Figure S1). However, two other chloroplast sequences that we obtained from the Reutlingen *Wellerhölzer* also date back to the 14th century (KP210196 and KP210197, respectively) and these have a lower number of SNPs compared to all other samples (44 SNPs). Hence, although the low sampling size does not allow conclusive analyses, our analyses show that genetic variation over time has been maintained in rye fields up till the 19th century as illustrated by the variation that we were able to recover in the samples from Zaisenhausen/Mulfingen.

### Genetic diversity in *Wellerhölzer* rye is reduced with the intensification of crop cultivation

To gain insights into the evolutionary trajectory of the medieval cereals with specific focus on the medieval rye, we obtained 17 complete chloroplast sequences of historical cereals extracted from the walls of *Wellerhölzer*. We used these sequences to address their phylogenetic relationships with 17 other chloroplast sequences of present-day samples (Figure 2B). *Wellerhölzer* samples identified as wheat (KP210077) and barley (KP210078) were grouped with the respective present-day accessions of these two species, thereby confirming the correct archaeobotanical identification. Chloroplast sequences of non-crop *Secale* species^29^ (Table S3) clustered together, while the historical *Wellerhölzer* chloroplast sequences, including those identified as Cerealia (i.e., cereals not identified to genus or species level) were clustered together, but also show the higher relatedness to the cv. Imperial than to the non-crop *Secale* species. Among the *Wellerhölzer* samples, the phylogenetic analysis clustered samples from the same or near locations together (e.g., *Wellerhölzer* from Schwäbisch Hall and Weipertshofen), thereby reflecting closer genetic relatedness of these samples. However, some samples from the same location did not cluster together suggesting extensive variation in rye populations, not only within but also between locations. For example, *Wellerhölzer* sample KP210099 from Zaisenhausen/Mulfingen showed higher similarity in the chloroplast sequence to a *Wellerhölzer* sample from Göttingen than to a sample from the geographically much closer Schwäbisch Hall. In summary, the *Wellerhölzer* rye/Cerealia form a separate cluster from the non-crop Secale species, they show higher genetic relatedness to the modern cultivar Imperial and the genetic relatedness of these historical samples are in most cases location-dependent.

### Genetic variants in historical samples are most abundant in chloroplast genes related to energy metabolism and chloro-respiration

To further investigate the genetic diversity in chloroplast sequences of the historical rye samples, we characterized the distribution of variable sites across the chloroplast genomes and its genetic elements.

**Figure 3:**
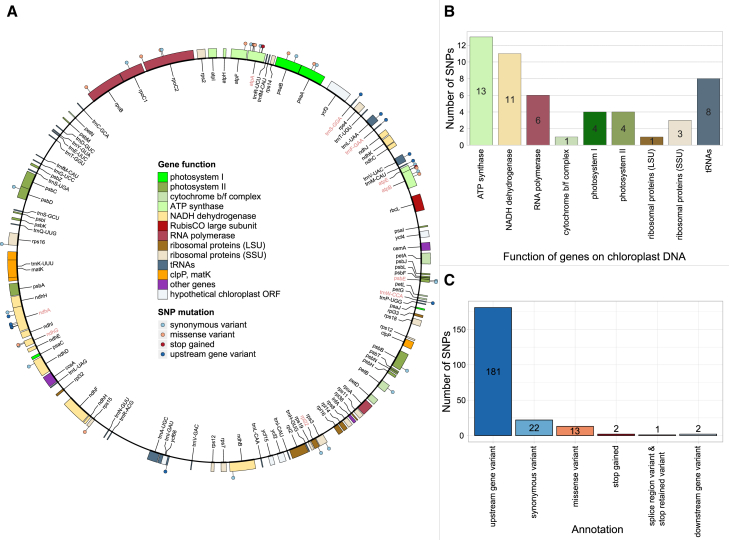
Mutation map in the chloroplast genomes of the historical rye samples (A) Distribution of the identified ancient mutations in chloroplast genes from rye varieties sampled from the medieval *Wellerhölzer*. “Lollipop” symbols designate single nucleotide polymorphisms (SNPs) present in respective rye chloroplast genes. Different colors of lollipops designate their functional annotation and gene names. (B) Bar plot displaying SNP number per 1000 bp window for all chloroplast genes that contain an SNP. The color of the bars designates the chloroplast gene function and corresponds to the legend in A. (C) Bar plot showing the number of all ancient SNPs in the chloroplast genome (inside and outside chloroplast genes) with their respective functional annotation identified by SnpEff.

Correlating positions of coding sequences and variable positions, we find that genetic variation is not randomly distributed across the chloroplast genomes. Of the total 217 SNPs we find 51 SNPs (23.5%) located in 28 out of the 107 annotated chloroplast genes (26.2%). Of these, 24 SNPs (47%) reside in genes involved in electron transport during photosynthesis and chloro-respiration, 13 SNPs (25.5%) in ATP synthase genes and 11 SNPs (21.5%) in NADH dehydrogenase-like (ndh) genes (Figures 3A and 3B). We note that of the 217 variable sites, four represent multiple alleles which we defined individually according to their putative impact, i.e., the total number of annotated SNPs was 221. We further assessed the putative relevance of the called SNPs and annotated their impact as “neutral” or “non-neutral”. The functional annotations of variants conducted with SnpEff^30^ showed that the identified SNPs were predominantly located in upstream regions of coding sequences including 181 (82%) (Figure 3C). Two SNPs (1%) were located downstream of gene sequences and 22 SNPs (10%) were neutral synonymous variants. Thirteen SNPs (6%) were missense, putatively non-neutral, variants, out of which seven alone were located in the genes encoding the enzymes ATP synthase (atpA, atpB, atpE, atpE, and atpF) and NADH dehydrogenase-like (ndhG, respectively). Additionally, two stop codon mutations (1%) were also present in ATP synthase genes (atpA and atpE), suggesting that the genes involved in cellular metabolism do not only possess higher mutation load in comparison to other chloroplast genes, but harbor higher number of non-neutral polymorphisms.

**Figure 4:**
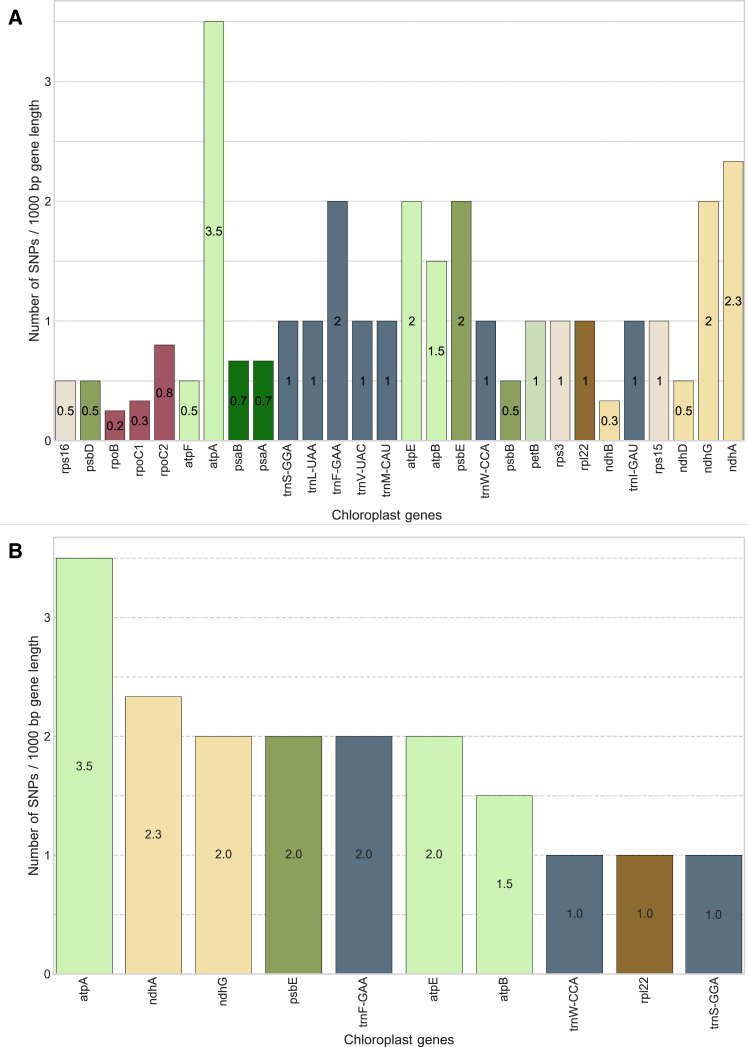
Normalized number of ancient SNPs in rye chloroplast genes (A) Number of SNPs per 1000 bp window for each rye chloroplast gene that harbored a mutation. (B) Barplot displaying the distribution of SNP numbers in ten rye chloroplast genes with the highest SNP load. Different bar colors in A and B correspond to the respective gene function of a gene carrying a mutation.

Next to recording the total number of SNPs in specific genes, we also calculated the SNP density per gene length (Figure 4). In agreement with our conclusion from crude SNPs numbers, we find that the genes with the highest frequency of variable sites, indeed, are genes involved in energy metabolism and chloro-respiration. ATP synthase A (atpA) and NADH dehydrogenase-like A (ndhA) had the highest densities of variable sites (3.5 and 2.3 SNPs per 1000 bp, respectively), while the Rubisco gene and mating type genes (clpP, matk) on the other hand, did not exhibit any variable sites at all. Taken together, our molecular analysis shows the highest variation at the chloroplast DNA level of the historical *Wellerhölzer* rye samples in genes involved in energy production and chloro-respiration, indicating the significant role that these genes might have had in the evolutionary trajectory of rye.

## Discussion

In our study, we made use of unique historical material originating from half-timbered houses at different locations in southern and central Germany to study genetic diversity in historical plant material. The buildings were constructed at different times from the 14th to the 19th centuries, using rye straw and its weedy accompanies for the manufacture of *Wellerhölzer*. The plant remains coiled around the wooden plank were largely incapsulated in the loam covering the *Wellerhölzer* for centuries, and are therefore well-preserved as desiccated, dry matter and thus intact. This allowed us to combine archaeobotanical analyses with ancient DNA sequencing methods to characterize the plant and rye diversity at field sites 200–600 years ago in today’s Germany.

Due to the excellent preservation of the rye straw associated with the *Wellerhölzer*, we were able to sequence the chloroplast genomes of seventeen samples, consisting mainly of samples of rye, but also one sample of barley and one of wheat (Figure 2B). Ancient DNA sequencing of plant material is challenged by the rapid decomposition of plant cell wall structures, which readily occurs under normal environmental conditions. The loam covering the straw has, however, resulted in a desiccated preservation of plant remains, which also sufficiently preserved the plant cell wall structures required for ancient DNA sequencing.

Studies of desiccated plant material from archaeological contexts have previously been conducted successfully on, among others, grains of free-threshing wheat (*Triticum aestivum* and *Triticum durum*), broomcorn millet (*Panicum miliaceum*), barley (*Hordeum vulgare*) and sorghum (*Sorghum bicolor)*.^31^^,^^32^^,^^33^^,^^34^ In the case of barley, 6000-year-old specimens were preserved inside a cave in the Judean desert aided by the hot and arid conditions of the surroundings.^32^ For this ancient barley material, exome capture data from six grains provided unique insights into the genetic composition of barley that was cultivated by Neolithic farmers. Intriguingly, comparing genetic information from a large number of modern barley varieties showed that already in the Neolithic, domesticated barley was considerably distinct from wild barley. This suggests a significant genetic modification already from the onset of barley domestication in the Fertile Crescent.

In contrast to the 6000-year-old barley remains, we find a significant variation in the chloroplast genome of the historical rye samples from today’s Germany. We mapped sequencing reads to the reference chloroplast genome of a modern rye variety *S. cereale* cv. Imperial,^35^ and identified 217 variable sites among the historical rye samples. The historical samples show higher relatedness to the cultivar Imperial than to non-domesticated *Secale* species reflecting the early signatures of domestication. We note, moreover, that Imperial was selected from the rye landrace “Schlanstedt” which originates from central Germany, i.e., a locality close to the origin of the historical rye samples described here. Among the historical samples, the samples from Schwäbisch Hall showed a large number of unique variable sites in the chloroplast genome, although these samples are among the more “recent” samples (ca year 1750). The identified variation in chloroplast sequences of historical rye likely reflects genetic variation that has been lost in the past century with the intensification of rye breeding. From the Medieval period onwards (ca. 8th century CE) and throughout the 19th century, the three-field system became a common cultivation practice within the modern borders of Germany.^19^ Hereby, one part is sown with a summer crop, another part is sown with a winter crop, the final part is left fallow, and each part is rotated every season.^19^^,^^36^ Using the moldboard plow, wet autumn soils were turned, enabling the cultivation of winter crops such as rye.^19^^,^^36^ Until the 1850’s, local selection processes resulted in a diverse set of varieties of rye, while from the 1850’s onward, selection processes targeted at specific characteristics of rye became more generalized, for example, leading to the first variety of rye that was more resistant to lodging.^2^ Our archaeobotanical analysis suggests that the rye from all investigated locations were likely cultivated as part of a three-field system. Based on the high genetic diversity of the samples included in our investigation, we can speculate that the samples represent landraces of rye that were the result of local selection processes, rather than targeted breeding. Future studies should include chloroplast sequences from a larger diversity of present-day rye cultivars and landraces to further dissect the extent and nature of lost variation during rye improvement.

We used the sequence data to investigate the distribution of historical genetic variants in relation to the chloroplast gene annotation. Our results show an increased number of genetic variants in the historical samples in the chloroplast genes that code for subunits of large protein complexes involved in energy production and chloro-respiration, primarily the ATP synthase and the NADH dehydrogenase-like (ndh) protein complex. The chloroplast ATP synthase produces the ATP needed for photosynthesis and plant growth,^37^ and the distinct haplotypes observed in the ATP synthase genes of the *Wellerhölzer* rye potentially reflect later breeding efforts to increase crop biomass.^38^ Furthermore, the chloroplastic ATP synthase complex also plays a role in plant stress response, since polymorphisms in the subunits of this complex have been associated with cold stress tolerances in cucumber.^39^ The chloroplast gene with the highest number of genetic variants, atpA, was found to be associated with increased resistance to the fungal pathogen *Botrytis cinerea* in tomato.^40^ ATP synthase genes are in general considered to play a significant role in plant stress tolerance and may, therefore, also have evolved in response to distinct environmental conditions and stressors. Chloroplast genes coding for subunits of the NDH complex involved in cyclic electron transport for photosystem I (PSI) and chlororespiration, also exhibited an increased number of genetic variants in the historical rye samples. The role of the NDH subunit encoding genes in abiotic stress alleviation has been demonstrated in several important crop species, such as tobacco and rice, where specific mutations in different NDH subunits were associated with improved response to heat and cold stress,^41^ as well as to attenuation of oxidative stress under fluctuating light conditions.^42^ Taken together, the highest portion of the identified point mutations in the chloroplasts of historical *Wellerhölzer* rye locate in genes involved in abiotic and biotic stress responses, and the distribution of these mutations in ancient chloroplast genomes possibly reflects non-targeted footprints of rye domestication and breeding.

In summary, our study demonstrates the great potential of combining aDNA sequencing and archaeobotanical analyses to address the breeding and cultivation history of crops. We here describe the plant diversity in rye fields at five rural locations in Germany. Our sampling include specimen from the 14th to the 19th centuries and illustrates a considerably change in biodiversity and genetic composition of rye landraces. This observation is in line with the development of agricultural practices and breeding efforts. Present day and future crop production is challenged by the consequences of climate change, including drought stress, flooding, and altered spectra of pathogens. Breeding efforts should aim to restore and preserve genetic variation in crops to increase resilience and adaptability of plant populations. In this regard, insights into the past spectrum of genetic diversity in cereal crops may point to ancient stress resistances that can be reestablished in present day crops to meet future challenges.

### Limitations of the study

Our reconstruction of past genetic variation in rye relied on sequencing of historical rye material obtained from several half-timbered buildings in Germany. A limitation of our study has been the availability of material as well-preserved “Wellehölzer” are rare. Therefore, our sampling of historical rye material includes individual samples from different historical times. In our comparative analyses, we have not been able to include “replicates” from different geographical sites representing the same historical time frame. Such sampling restrictions cannot be circumvented in studies involving historical material.

We focused our analyses on the chloroplast genome. This allowed us to compare a high-quality plastid genome sequence to compare genetic variation among rye accessions. Many domestication-related traits are encoded by the nuclear genome which was not analyzed here. With our ancient DNA sequencing approach, we were not able to obtain high quality data for the nuclear rye genome. Thereby we have not been able to present analyses of genetic variation in genes which may have been under selection during genetic improvements of rye in the past centuries.

## Resource availability

### Lead contact

Further information and requests regarding the analyzed ancient plant material should be directed to Sofia Filatova (sonja.filatova@rug.nl) and Wiebke Kirleis (wiebke.kirleis@ufg.uni-kiel.de).

### Materials availability

The "Wellehölzer" material which was used in this study for ancient DNA sequencing, is available from the lead contact with a completed materials transfer agreement.

### Data and code availability

Raw sequence data obtained from historical plant remains are available in zenodo repository (https://doi.org/10.5281/zenodo.12655083). Code used for the SNP calling, nucleotide diversity analysis and data visualization is available in Data S1 file.

## Acknowledgments

This work was funded through the 10.13039/501100001659DFG-funded Excellence Cluster ROOTS (EXC2150). We are grateful for support by PD Dr. Elena Marinova and Dr. Elske Fischer from the Archaeobotanical Laboratory of the State Office for Cultural Heritage Baden Württemberg, Hemmenhofen/Gaienhofen, Germany.

## Author contributions

J.K.: Conceptualization, formal analysis, visualization, writing – original draft; E.H.S.: Conceptualization, writing and editing of the manuscript, project management; S.F.: Conceptualization, writing and editing of the manuscript; W.K.: Conceptualization, writing and editing of the manuscript; F.S.: Writing and editing of the manuscript; B.K.-K.: Conceptualization, data generation, editing of the manuscript; B.C.: Data generation; M.R.: Data generation, editing of the manuscript.

## Declaration of interests

The authors declare no conflicts of interests.

## STAR★Methods

### Key resources table

**Table:** 

REAGENT or RESOURCE	SOURCE	IDENTIFIER
**Biological samples**		

*Triticum aestivum* ssp. *aestivum -* ear fragments	Göttingen	KP210077
Hordeum vulgare ssp. vulgare - rachis fragments	Göttingen	KP210078
Secale cereale - ear	Göttingen	KP210080
Cerealia indet - straw fragments	Göttingen	KP210082
Cerealia indet - straw fragments	Göttingen	KP210083
Cerealia indet - straw fragments	Göttingen	KP210084
Cerealia indet - straw fragments	Göttingen	KP210086
Secale cereale L. - ear fragment	Zaisenhausen	KP210099
Secale cereale L. - ear fragment	Weipertshofen	KP210124
Secale cereale L. - ear fragment	Schwäbisch Hall	KP210160
Secale cereale L. - ear fragment	Schwäbisch Hall	KP210161
Secale cereale L. - ear fragment	Schwäbisch Hall	KP210169
Secale cereale L. - ear fragment	Schwäbisch Hall	KP210170
Secale cereale L. - ear fragment	Schwäbisch Hall	KP210171
Secale cereale L. - ear fragment	Reutlingen	KP210195
Secale cereale L. - ear fragment	Reutlingen	KP210196
Secale cereale L. - ear fragment	Reutlingen	KP210197

**Deposited data**		

Raw sequence data	https://doi.org/10.5281/zenodo.12655083	https://doi.org/10.5281/zenodo.12655083

**Software and algorithms**		

nf-core/EAGER v2.4.7	Peltzner et al.^43^	https://nf-co.re/eager/2.4.7/
samtools (version 1.7)	Danecek et al.^44^	http://www.htslib.org/
bcftools (version 1.7)	Danecek et al.^44^	https://samtools.github.io/bcftools/bcftools.html
tabix (version 1.10.2)	Danecek et al.^44^	http://www.htslib.org/doc/tabix.html
SnpEff (version 5.1day)	Cingolani et al.^45^	http://pcingola.github.io/SnpEff/
bedtools (v2.26.0)	Quinlan and Hall^46^	https://bedtools.readthedocs.io/en/latest/
ClustalW 2.1	Thompson et al.^47^	https://bioweb.pasteur.fr/packages/pack@ClustalW@2.1
SeaView (version 5.0.5)	Gouy et al.^48^	https://doua.prabi.fr/software/seaview
Figtree (v1.4.4)		http://tree.bio.ed.ac.uk/software/figtree/
vcftools (version 0.1.15)	Danecek et al.^49^	https://vcftools.sourceforge.net/
Python3 and R script for analysis and visualization	This paper	Data S1

### Experimental model and study participant details

#### Plant material

Plant material used in this study consisted of 17 samples of historical plant material from construction parts of half-timbered houses (plant-tempered clay, with focus on *Wellerhölzer*) built between the 14^th^ and 19^th^ century from five locations in Germany (Figure 1). Seven *Wellerhölzer* samples were collected from the old town hall in Göttingen, and the remaining ten historical samples were obtained from the south German half-timbered houses in Schwäbisch Hall (five samples), Reutlingen (three samples), Zaisenhausen/Mulfingen (one sample) and Weipertshofen (one sample) (Figure 1). According to available historical data, for the buildings from which the material was collected, the plant material isolated from these locations, dates from 14th to 19th century according to available historical data for the buildings from which the material was collected.^24^^,^^26^^,^^27^ We note that radiocarbon dates taken on material dating between 1650 and AD 1950 unfortunately cannot be established with certainty due to the Maunder Minimum and the Suess effect, which greatly impacted the 14C content in the atmosphere.^50^^,^^51^ Radiocarbon dates falling within the scope of these atmospheric events results in multiple possibilities and can thus only be indicated broadly. In this study, we therefore limit ourselves to the construction dates of the buildings, being aware that the actual dates of the plant remains may be younger due to later reconstruction activities. A table listing the samples, their origin and 14C dates is provided in Table S1.

### Method details

#### Archaeobotanical analysis

Archaeobotanical analyses of the plant material was performed by different individuals at different occasions. The material from Schwäbisch Hall, Reutlingen and Zaisenhausen/Mulfingen was analyzed by Rösch and Fischer,^24^^,^^27^^,^^28^ the material from Weipertshofen by Fischer and Rösch the material from Göttingen by Filatova et al.^25^ The plant remains were separated from the sediment matrix by soaking in water and subsequently sieving the material using (stacked) sieves with mesh widths varying from 2 mm to 0.25 mm. The remains were dried before being analyzed using low-magnification (up to 40 x) stereo microscopes. Cultivation practices and soil conditions of the rye used in the two *Wellerhölzer* from Göttingen and Schwäbisch Hall were reconstructed based on a combination of autecology and functional autecology of the presumed arable weed taxa (for a more elaborate discussion, see^25^). These two approaches are based on the notion that characteristics of plants are indicative of certain climatic conditions (e.g., temperature and light), soil conditions (e.g., pH and nitrogen), and activities of soil disturbance (e.g., digging and trampling).^24^^,^^52^^,^^53^^,^^54^^,^^55^

Taxa that were identified to species level were included in the analysis. Functional attributes and autecological characteristics of the selected species were used to reconstruct cultivation practices (Appendix 1; Table S2). Data on synecology, autecology, and functional attributes were collected from previous works.^55^^,^^56^^,^^57^

#### Sample preparation, DNA extraction and sequencing

The historical plant material used in this study included straw, spikelets, and ears of cereals which were identified to belong to rye as well as wheat and barley based on visual examination of morphological traits (Table S1). To prepare selected rye samples for the DNA extraction, approximately 0.1–0.2 g of desiccated rye material of each sample was washed in a 2% sodium hypochlorite (NaOCl) solution, followed by an additional ethanol (C_2_H_5_OH) wash for the surface cleaning from contaminants.

Ancient DNA was extracted according to a previously published protocol specifically developed for the extraction of ancient DNA using N-phenacyl thiazolium bromide (PTB) with minor modifications.^58^ In short, the washed plant remains were frozen with dry ice and grinded, overnight incubation at 37°C in a buffer containing N-phenacyl thiazolium bromide (2.5 mM PTB, 50 mM dithiothreitol (DTT), 10 mM TRIS, 10 mM ethylenediaminetetraacetic acid (EDTA), 5 mM sodium chloride (NaCl), 1% sodium dodecyl sulfate (SDS), 0.4 mg/mL Proteinase K) in a rotator. After incubation, DNA was extracted from and converted into partial Uracil-DNA glycosylase (UDG) libraries ^59^⁠, following the established laboratory guidelines for aDNA work.^43^⁠

Sequencing was performed on the Illumina NovaSeq6000 platform at the Institute of Clinical Molecular Biology, Kiel University, Germany using the NovaSeq S1 Kits with 200 cycles.

Illumina reads were deposited at https://doi.org/10.5281/zenodo.12655083.

#### Read mapping, variant calling and annotation

In our study, we focused on the chloroplast genome that occurs in higher copy numbers than the nuclear genome, which congruently is reflected in sequence abundance. To assess the nucleotide diversity of the chloroplast genome between the modern and historical rye, Illumina paired reads from *Wellerhölzer* rye samples were mapped to the reference chloroplast genome of rye, the cultivar Imperial here specified with the code KC912691^35^ with the nf-core/EAGER, bioinformatic pipeline specifically tailored for the computational analysis of ancient DNA.^44^ The same approach for read mapping and variant calling was conducted for the particular Wellerhölzer samples that we identified as wheat (KP210077), barley (KP210078) and Cerealia (KP210082, KP210083, KP210084, KP210086). To this end, reads obtained from these Wellerhölzer samples were mapped to the KC912694 reference chloroplast genome of wheat, and to the KC912687 reference chloroplast genome of barley, respectively,^35^ while the *Wellerhölzer* Cerealia samples were mapped to the reference chloroplast genome of rye. The wheat, barley and Cerealia sequences were subsequently used for the phylogenetic analyses (see below).

We assessed the aDNA damage mapping profiles via the mapDamage tool incorporated in the nf-core/EAGER pipeline,^44^ in order to detect specific nucleotide misincorporations and DNA fragmentation signatures in sequencing reads, including an increase of C→T and G→A substitutions at the 5′ and 3′ regions of the sequenced DNA, a typical signature in degenerated ancient DNA.^49^ Mapped reads from which duplicates were removed by the EAGER pipeline and which otherwise passed the mapping-damage-control and quality check, were further used for the variant calling. Genetic variants from the chloroplast genome of medieval cereals were called by samtools mpileup (version 1.7, options -C 50 -Q 0 -q 20 -uf), followed by bcftools call (version 1.7, options -vc -O u -o).^45^ For our analyses, we have focused exclusively on biallelic SNPs. Therefore, called variants were filtered for biallelic SNPs with bcftools view, and only SNPs with mapping quality higher than 20 were used for the downstream analyses.

To perform functional annotation of the SNPs identified in the *Wellerhölzer* rye chloroplast genome, vcf (variant call format) files from all *Wellerhölzer* rye samples were merged into a single vcf file via vcf-merge program from vcftools (version 0.1.15).^46^ The merged vcf file was further used as an input for SnpEff (version 5.1day), a program for annotating and predicting the SNP effects.^30^ The number of alleles with a specific annotation was calculated from the output SnpEff file with a custom-made Python script. Lastly, the number of SNPs per specific chloroplast gene was calculated with a custom Python script from an output file from bedtools intersect (v2.26.0, options -wa -wb)^60^ between the gene annotation file of the rye reference chloroplast genome and bed file with the coordinates of SNPs identified in *Wellerhölzer* rye samples. The SNP number per chloroplast gene was normalized for gene size and calculated per 1000 bp windows with a custom-made Python script. The custom-made scripts used for the analyses and the plotting of figures are provided in Data S1.

#### Chloroplast sequence variation and phylogenetic analysis

To characterize the chloroplast sequence variation between the *Wellerhölzer* samples, we identified all genetic variants and distinguished the variable sites specific to certain sampling sites or shared across different sampling sites (Figures 2A and S1). Further, for phylogenetic analysis, we generated a Neighbor-Joining tree that included the chloroplast genome of rye cv. Imperial^35^ and chloroplast sequences of the historical *Wellerhölzer* samples, as well as the chloroplast sequences of rye relative species and rye subspecies. For the genus *Secale*, thirteen chloroplast sequences from eight additional rye relative species and subspecies^29^^,^^61^^,^^62^^,^^63^ were included in the Neighbor-Joining analysis*,* as well as the reference chloroplast sequences of *Triticum aestivum* and *Hordeum vulgare*^47^^,^^64^ (Figure 2B).

For the chloroplast sequences of the historical *Wellerhölzer* rye and Cerealia samples, we generated consensus sequences using the reference rye chloroplast sequence KC912691^35^ and the vcf file of a respective historical sample via bcftools consensus.^45^ For the two chloroplast sequences of wheat and barley, a consensus sequence was generated with the respective reference chloroplast sequence of wheat and barley, respectively.^35^ Multiple sequence alignments between were constructed using ClustalW^48^ which were subsequently used to generate a Neighbor-Joining tree (using 1000 bootstraps) with the SeaView software.^65^ Trees were visualized using Figtree (v1.4.4) (http://tree.bio.ed.ac.uk/software/figtree/).

### Quantification and statistical analysis

Calculating the number of SNPs per 1000 bp was done with 03_SNP_per_gene.sh bash script provided in Data S1. Additional statistical analyses were not performed.

## Supplemental information

Document S1. Figures S1, Tables S1–S3, and Appendix 1

Data S1. Scripts used for sequence analyses
