# Supplementary material for: Sequencing of historical plastid genomes reveal exceptional genetic diversity in early domesticated rye plants
Source: iScience. 2025 Jun 6;28(7):112716. doi: 10.1016/j.isci.2025.112716 (PMC12269287; doi:10.1016/j.isci.2025.112716)
Supplement: Document S1. Figures S1, Tables S1–S3, and Appendix 1 [file mmc1.pdf]

iScience, Volume *28*

Supplemental information

Sequencing of historical plastid genomes reveal exceptional genetic diversity in early domesticated rye plants

Jovan Komluski, Sofia Filatova, Frank Schlütz, Benjamin Claaßen, Manfred Rösch, Ben Krause-Kyora, Wiebke Kirleis, and Eva H. Stukenbrock

Fig. S1. Intersections between ancient SNPs from historical rye samples, related to Fig. 2A.

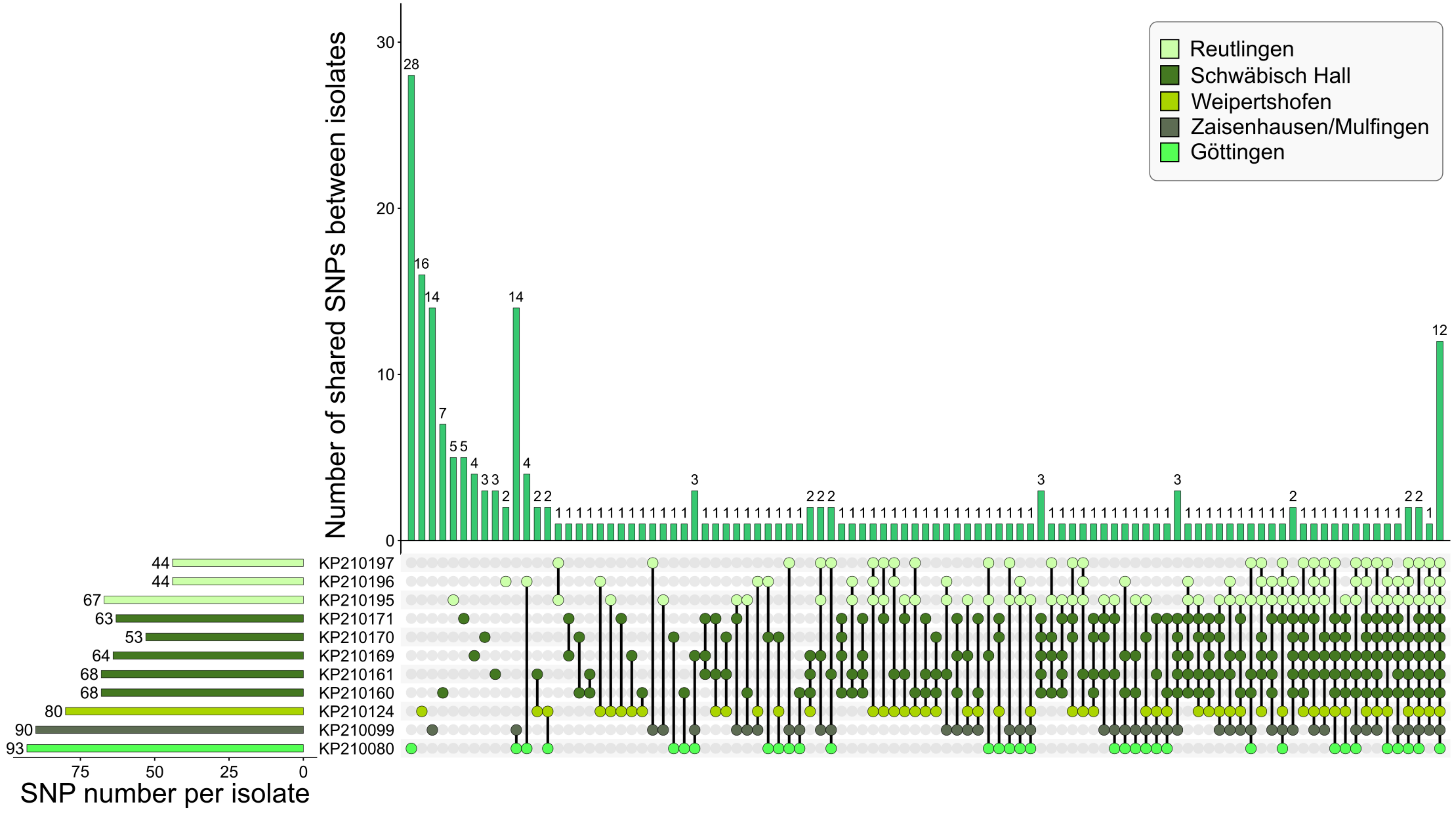

Upset plot showing shared ancient SNP between historical rye samples from different sampling locations. Different shades of green correspond to sampling location.

Table S1. List of ancient cereal varieties with their 14C dating used in this study, related to Fig. 1 and Fig. 2.

aDNA ID	Taxon	Material (n)	Germany	Town	Address	Component	GVAC-ID	subsampel
KP210077	*Triticum aestivum ssp. aestivum*	Ear fragments (2)	middle					a
KP210078	*Hordeum vulgare ssp. vulgare*	Rachis fragments (20)	middle					b
KP210080	*Secale cereale*	Ear (1)	middle					g
KP210082	*Cerealia indet*	Straw fragments***	middle	Göttingen	Markt 8, old town hall	Wellerholz	6	i
KP210083	*Cerealia indet*	Straw fragments***	middle					j
KP210084	*Cerealia indet*	Straw fragments***	middle					k
KP210086	*Cerealia indet*	Straw fragments***	middle					m
KP210099	*Secale cereale L.*	Ear fragment (1)	south-west	Zaisenhausen*	House No. 35	clay wickerwork**	22	d
KP210124	*Secale cereale L.*	Hull (1)	south-west	Weipertshofen*	Käshof Wiler, Käsbach	Wellerholz	23	l
KP210160	*Secale cereale L.*	Ear fragment (1)	south-west					c
KP210161	*Secale cereale L.*	Ear fragment (1)	south-west					d
KP210169	*Secale cereale L.*	Ear fragment (1)	south-west	Schwäbisch Hall	Pfarrgasse 9	Wellerholz	27	l
KP210170	*Secale cereale L.*	Ear fragment (1)	south-west					m
KP210171	*Secale cereale L.*	Ear fragment (1)	south-west					n
KP210195	*Secale cereale L.*	Ear fragment (1)	south-west					a
KP210196	*Secale cereale L.*	Ear fragment (1)	south-west	Reutlingen	Pfäfflinshofstraße 4	clay wall	30	b
KP210197	*Secale cereale L.*	Ear fragment (1)	south-west					c

* replaced to open air museum in Wackershofen
**Lehmgeflecht
***several

Sample ID	**building**	**14C ID**	**cal CE (95%)**	***Comments***
GVAC_06_a				Two ear fragments of bread wheat: first ear = 6 rachis nodes + 3 spikelets; second ear = 4 rachis nodes + 3 spikelets
GVAC_06_b				Rachis fragments with a total of 20 rachis nodes
GVAC_06_g				One ear consisting of 19 spikelets and including 2 fruiting bodies of ergot
GVAC_06_i	14th cent.	KIA-55740	1640-1946	One vial full with straw fragments with black lesions, possibly fungus.
GVAC_06_j				One vial full with straw fragments with black lesions, possibly fungus.
GVAC_06_k				One vial full with straw fragments with black lesions, possibly fungus.
GVAC_06_m				One vial full with straw fragments with black lesions, possibly fungus.
GVAC_22_d	19th cent.	KIA-56310	1658-1947	Samples from the inside of the partition wall on the "Stube" on the second floor of the house
GVAC_23_l	1585 CE	KIA-56312	1663-1946	Wellerholz originally located above the kitchen
GVAC_27_c				Wellerholz originally located in the ceiling of the ground floor
GVAC_27_d				Wellerholz originally located in the ceiling of the ground floor
GVAC_27_l	~1750 CE	KIA-56316	1649-1948	Wellerholz originally located in the ceiling of the ground floor
GVAC_27_m				Wellerholz originally located in the ceiling of the ground floor
GVAC_27_n				Wellerholz originally located in the ceiling of the ground floor
GVAC_30_a				-
GVAC_30_b	1337 CE	-	-	-
GVAC_30_c				-

Table S2. Overview of autecological characteristics and functional attributes collected for the taxa of arable weeds and wild plants from the Wellerholz from Schwäbisch Hall.

Taxon original	Absolute quantity	Plant community nr.	Plant community	Lifeform	Lifespan	Flowering start (month)	Flowering end (month)	Flowering duration	Flowering onset class	Flowering duration class	Reproduction type	Vegetative reproduction	Ecological strategy	Floristic status	Water value	Reaction value	Nitrogen value
Agrostemma githago	1	3.4	Secalietea	Therophyte, Hemicryptophyte	Annual	6	7	2	Intermediate	Short	seed	-	Competitors/ ruderals	Archaeophyte	indiff.	indiff.	1
Alopecurus myosuroides	17	3.4	Secalietea	Hemicryptophyte, Therophyte	Annual	5	10	6	Intermediate	Long	seed	-	Ruderals	Archaeophyte	5	7	6
Apena spica-venti	33	3.4	Secalietea	Therophyte, Hemicryptophyte	Annual	6	7	2	Intermediate	Short	seed	-	Competitors/ ruderals	Indigenous	6	6	4
Chenopodium album	3	3.3	Chenopodietea	Therophyte	Annual	7	10	4	Late	Medium	seed	-	Competitors/ ruderals	Archaeophyte	4	indiff.	7
Fragaria vesca	1	6.2	Epilobietea	Hemicryptophyte	Pluriennial-pollakanthic	5	6	2	Intermediate	Short	seed/vegetative	Runner, rhizome	Competitors/ stress-tolerators-ruderals	Indigenous	5	indiff.	6
Matricaria chemomilla	1	3.4	Secalietea	Therophyte, Hemicryptophyte	Annual	5	8	4	Intermediate	Medium	seed	-	Ruderals	Archaeophyte	6	5	5
Myosodon aquaticum	2	3.5	Artemisietea vulgaris	Hemicryptophyte, Geophyte	Plurennial-pollakanthic	6	9	4	Intermediate	Medium	seed/vegetative	Runner	Competitors/ stress-tolerators	Indigenous	8	indiff.	8
Odontites vernus	3	3.4	Secalietea	Therophyte	Annual	5	7	3	Intermediate	Short	seed	-	Ruderals	Indigenous	?	?	?
Papaer rhoeas	1	3.4	Secalietea	Therophyte, Hemicryptophyte	Annual	5	7	3	Intermediate	Short	seed		Competitors/ ruderals	Archaeophyte	5	7	6
Poa annua	2	3.7	Plantaginetea majoris	Therophyte, Hemicryptophyte	Annual, plurennial-pollakanthic	1	12	12	Early	Long	seed/vegetative	Runner	Ruderals	Indigenous	6	indiff.	8
Polygonum aviculare	258	indiff.	Indiff	Therophyte	Annual	5	11	7	Intermediate	Long	seed	-	Ruderals	Indigenous	indiff.	indiff.	indiff.
Rubus idaeus	1	indiff.	Indiff	Pseudophanerophyte	Pluriennial-pollakanthic	5	6	2	Intermediate	Short	seed/vegetative	Root shoot	Competitors	Indigenous	indiff.	indiff.	6
Senecio sylvaticus	1	6.2	Epilobietea	Hemicryptophyte, Therophyte	Annual	6	8	3	Intermediate	Short	seed	-	Competitors/ ruderals	Indigenous	5	5	8
Senacio vulgaris	2	3.3	Chenopodietea	Hemicryptophyte, Therophyte	Annual	2	11	10	Early	Long	seed	-	Ruderals	Indigenous	5	indiff.	8
Sonchus oleraceus	13	3.3	Chenopodietea	Therophyte, Hemicryptophyte	Annual	6	10	5	Intermediate	Medium	seed	-	Competitors/ ruderals	Indigenous	4	8	8
Stellaria media	1	3.3	Chenopodietea	Therophyte, Hemicryptophyte	Annual	1	12	12	Early	Long	seed	-	Competitors/ ruderals	Archaeophyte	4	7	8

Table S3. List of chloroplast genomes of modern cereal varieties and rye accessions that were used for phylogenetic analysis, related to Fig. 2B.

**Accession Number**	***Species***	**Sequence type**	***Species label on phylogenetic tree (Fig. 2B)***	**NCBI**	**Publication**
MZ507427.1	*Secale cereale subsp. segetale*	chloroplast genome	*Secale cereale ssp segetale A*	https://www.ncbi.nlm.nih.gov/nuccore/MZ507427.1	https://doi.org/10.1080/23802359.2022.2080600
OL688773.1	*Secale cereale subsp. segetale*	chloroplast genome	*Secale cereale ssp segetale B*	https://www.ncbi.nlm.nih.gov/nuccore/OL688773.1/	Direct submission
KY636137.1	*Secale strictum*	chloroplast genome	*Secale strictum A*	https://www.ncbi.nlm.nih.gov/nuccore/KY636137.1/	https://doi.org/10.1186/s12862-017-0989-9
KY636138.1	*Secale strictum*	chloroplast genome	*Secale strictum B*	https://www.ncbi.nlm.nih.gov/nuccore/KY636138.1	https://doi.org/10.1186/s12862-017-0989-9
LC645210.1	*Secale strictum kuprijanovii*	chloroplast genome	*Secale strictum kuprijanovii*	https://www.ncbi.nlm.nih.gov/nuccore/LC645210.1/	DOI: 10.1080/23802359.2021.2011447
OL979486.1	*Secale strictum subsp. strictum*	chloroplast genome	*Secale strictum ssp strictum*	https://www.ncbi.nlm.nih.gov/nuccore/OL979486.1/	Direct submission
MW557517.1	*Secale sylvestre*	chloroplast genome	*Secale sylvestre A*	https://www.ncbi.nlm.nih.gov/nuccore/MW557517.1	https://doi.org/10.1007/s13353-021-00656-x
LC649171.1	*Secale sylvestre*	chloroplast genome	*Secale sylvestre B*	https://www.ncbi.nlm.nih.gov/nuccore/LC649171.1	Direct submission
KC912694.1	*Triticum aestivum*	chloroplast genome	*Triticum aestivum*	https://www.ncbi.nlm.nih.gov/nuccore/KC912694	https://doi.org/10.1371/journal.pone.0085761
KY636134.1	*Secale cereale voucher R 1027 (synonym Secale vavilovii)*	chloroplast genome	*Secale vavilovii A*	https://www.ncbi.nlm.nih.gov/nuccore/1209566456	https://doi.org/10.1186/s12862-017-0989-9
KY636132.1	*Secale cereale subsp. segetale Zhuk voucher PI 618671*	chloroplast genome	*Secale cereale ssp. segetale C*	https://www.ncbi.nlm.nih.gov/nuccore/1209566299	https://doi.org/10.1186/s12862-017-0989-9
KY636135.1	*Secale cereale voucher PI 253957 (synonym Secale vavilovii)*	chloroplast genome	*Secale vavilovii B*	https://www.ncbi.nlm.nih.gov/nuccore/1209566535	https://doi.org/10.1186/s12862-017-0989-9
KY636136.1	*Secale cereale subsp. rigidum Vavilov & Antropov voucher PI 618669*	chloroplast genome	*Secale cereale ssp. rigidum*	https://www.ncbi.nlm.nih.gov/nuccore/1209566613	https://doi.org/10.1186/s12862-017-0989-9
KY636133.1	*Secale cereale subsp. ancestrale Zhuk voucher PI 618665*	chloroplast genome	*Secale cereale ssp. ancestrale*	https://www.ncbi.nlm.nih.gov/nuccore/1209566377	https://doi.org/10.1186/s12862-017-0989-9
KC912687.1	*Hordeum vulgare*	chloroplast genome	*Hordeum vulgare*	https://www.ncbi.nlm.nih.gov/nuccore/KC912687	https://doi.org/10.1371/journal.pone.0085761
OQ700975.1	*Secale cereale subsp. vavilovii*	chloroplast genome	*Secale cereale ssp vavilovii*	https://www.ncbi.nlm.nih.gov/nuccore/OQ700975.1	Direct Submission
KC912691.1	*Secale cereale*	chloroplast genome	*Secale cereale Imperial KC912691.1*	https://www.ncbi.nlm.nih.gov/nuccore/KC912691.1/	https://doi.org/10.1371/journal.pone.0085761

Appendix 1: Archaeobotanical analyses

Methods

For the analysis of archaeobotanical remains obtained from the *Wellerholz* from Göttingen, including sample processing sorting and quantification, we used our previously established pipeline for the *Wellerhölzer* material (Filatova et al., 2021). The details on the analysis of archaeobotanical remains obtained from the Wellerholz from Schwäbisch Hall conducted by Manfred Rösch and Elske Fischer at the archaeobotanical department of the State Office for Cultural Heritage Baden Württemberg in Hemmenhofen/Gaienhofen (Germany) are published in Rösch and Fischer (1997).

Cultivation practices and soil conditions of the rye harvests represented in the two *Wellerhölzer* were reconstructed based on a combination of data on 1) the autecology and 2) the functional autecology of the identified arable weed taxa (for a more elaborate discussion, see Filatova et al. (2021)). These two approaches are based on the notion that characteristics of plants are indicative of certain climatic conditions (e.g., temperature and light), soil conditions (e.g., pH and nitrogen), and activities of soil disturbance (e.g., digging and trampling) (Bogaard et al., 1999; Charles et al., 2002; Duckworth et al., 2000; Ellenberg, 1988).

All taxa that were identified to species level were included in the analysis following a qualitative approach. Functional attributes and autecological characteristics of the selected species were used to reconstruct cultivation practices (Table A.1). Data on synecology, autecology, and functional attributes were collected from Ellenberg (1988) and Kühn et al. (2004).

Table A.1 Autecological characteristics and functional attributes used to indicate cultivation practice. The criteria used to define flowering onset and duration are: Early flowering onset: January-April; Intermediate flowering onset: April-June; Late flowering onset: July or later; Short flowering duration: 1-3 months; Medium flowering duration:

4-5 months; Long flowering duration: > 5 months. The table and flowering onset/duration criteria are adapted from Hillman 1981, Kreuz and Schäfer 2011, Neveu et al. 2021, Charles et al. 2002, Jones et al. 2005, Bogaard et al. 2005, Van der Veen 1992 and Leuschner and Ellenberg 2017.

**Cultivation practice**		**Associated (aut)ecological characteristic**
Sowing season	Autumn sowing	Early flowering onset
		Intermediate flowering onset
		Short flowering duration
		Long flowering duration
	Spring sowing	Late flowering onset
	Inconclusive	Medium flowering duration
Disturbance degree	High disturbance	Long flowering duration
		Ruderal strategy type
		Therophyte lifeform
		Annual with reproduction by seed
		Perennial with subterranean reproductive organs
	Low disturbance	Perennial without subterranean reproductive organs
		Short flowering duration
Ploughing season	Autumn ploughing	Early flowering onset and/or short flowering duration
	Spring ploughing	Late flowering onset

Results

The material from the *Wellerholz* from Schwäbisch Hall (dating to 1750 AD) comprised 829 plant remains. The majority were remains of cereals (51%; mainly rye ears) followed by arable weeds and wild plants (44%), the latter of which included 16 taxa that were identified to species level (Table A.2 and Table S3). Most of these species thrive in the modern segetal plant communities of the Chenopodietea (3.3 in Table S3)

and the Secalietea (3.4 in Table S3) as decribed in Ellenberg (1988). The dominant lifeform is therophyte-hemicryptophyte (n=10; Fig. A.1), followed by therophyte (n=3), hemicryptophye (n=1) and hemicryptophyte-geophyte (n=1). Twelve species have an annual life lifecycle, three a perennial one, and one species can thrive both as an annual as well as a perennial. An intermediate (n=12) flowering onset is most common, followed by early (n=3) and late (n=1), while a short flowering duration prevails (n=7), followed by long (n=5) and medium (n=4). Species that have both a competitive and a ruderal ecological strategy as well as species that solely have a ruderal strategy are equally represented (n=6), and two different combinations of competitors, ruderals and stress-tolerators occur in one occasion each.

Table A.2 Overview of plant taxa identified to the level of species from the Wellerholz from Schwäbisch Hall (Rösch and Fischer, 1997).

**Taxon original**	**Absolute find quantity**
Agrostemma githago	1
Alopecurus myosuroides	17
Apena spica-venti	33
Chenopodium album	3
Fragaria vesca	1
Matricaria chemomilla	1
Myosodon aquaticum	2
Odontites vernus	3
Papaer rhoeas	1
Poa annua	2
Polygonum aviculare	258
Rubus idaeus	1
Senecio sylvaticus	1
Senacio vulgaris	2
Sonchus oleraceus	13
Stellaria media	1

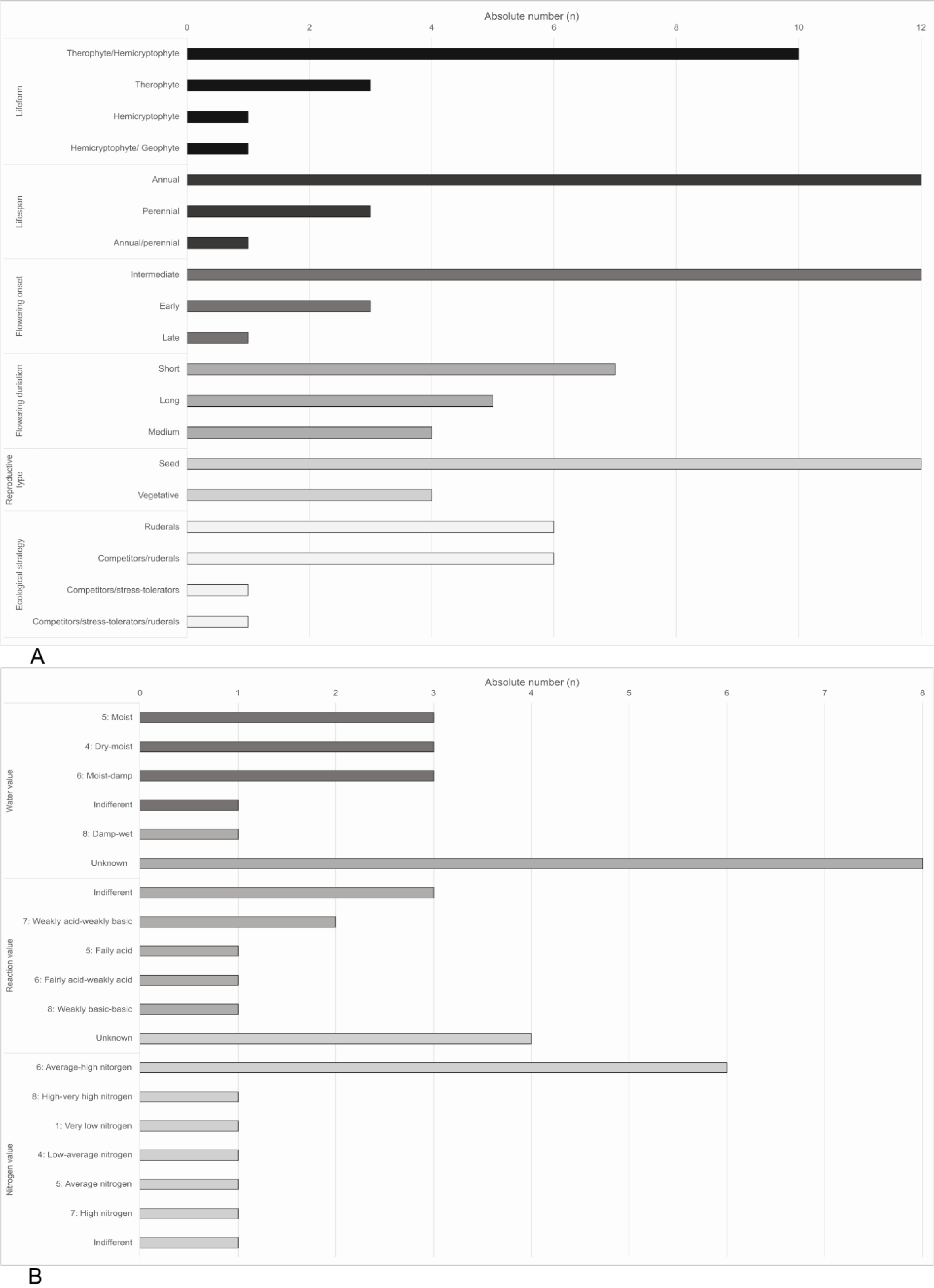
Absolute number (n)
0
2
4
6
8
10
12
Lifeform
Therophyte/Hemicryptophyte
Therophyte
Hemicryptophyte
Hemicryptophyte/ Geophyte
Lifespan
Annual
Perennial
Annual/perennial
Flowering onset
Intermediate
Early
Late
Flowering duration
Short
Long
Medium
Reproductive type
Seed
Vegetative
Ecological strategy
Ruderals
Competitors/ruderals
Competitors/stress-tolerators
Competitors/stress-tolerators/ruderals
A
Absolute number (n)
0
1
2
3
4
5
6
7
8
Water value
5: Moist
4: Dry-moist
6: Moist-damp
Indifferent
8: Damp-wet
Unknown
Reaction value
Indifferent
7: Weakly acid-weakly basic
5: Faily acid
6: Fairly acid-weakly acid
8: Weakly basic-basic
Unknown
Nitrogen value
6: Average-high nitorgen
8: High-very high nitrogen
1: Very low nitrogen
4: Low-average nitrogen
5: Average nitrogen
7: High nitrogen
Indifferent
B

Fig. A.1 Bar chart summarizing the autecological data and functional attributes of the arable weeds and wild plants from Schwäbisch Hall that were used to characterize the sowing time and cultivation intensity of the rye (the data collected from Ellenberg (1988) and Biolflor (Klotz et al. 2002). **b.** Bar chart summarizing the edaphic factors used to characterise conditions of the soil based on the arable weeds and wild plants from Schwäbisch Hall (values collected from Ellenberg 1988).

According to the criteria summarized in Table A.1, an early and intermediate flowering onset with a short and long flowering duration are typical indicators of autumn sowing. The prevalence of therophyte lifeforms with an annual reproduction cycle and a ruderal strategy type indicate a high level of disturbance, and the early flowering onset and short flowering duration imply autumn ploughing. The co-occurrence of nearly equal amounts of short and long flowering durations renders these two characteristics inconclusive with respect to the definition of the degree of disturbance. Taken together, these data strongly suggest that the rye was sown in autumn, and thus grew as a winter rye, and that the arable weeds and wild plants were adapted to a high degree of disturbance.

Ellenberg's indicator values of edaphic factors show a diverse range of values related to the arable weeds and wild plants from Schwäbisch Hall (Fig. A.b). The water value is dominated by moist indicators (n=5), followed by dry-moist (n=3), and moist-damp (n=3) indicators. One species thrives in damp-wet soils, while three are indifferent and one is unknown. The majority of the species are indifferent to soil pH (n=8), three are indicative of weakly acidic-weakly basic conditions, two of fairly acidic conditions, while fairly acidic-weakly acidic and weakly basic conditions are indicated by one species each. High to very high nitrogen content is indicated by six species, average to high nitrogen content is indicated by four species, and all other values (ranging from very low to high nitrogen content) are indicated by one species each. In summary, the indicator values suggest moist soil conditions, a high nitrogen content and more acidic than basic conditions. Reaction values show intermediate to acid conditions since many species are indifferent. Many indicate basic soils and species with indication for acid soils can grow on basic soils as well. The high representation of species growing on acid soils however may hint to originally poor soils with low pH that were manured. It has been proposed that, at least from the medieval period onward, rye was typically

grown on more acidic soils than species of wheat, which corresponds well to the conditions reflected by the indicator values (Rösch and Fischer, 1999). The relatively high nitrogen content might be indicative for manuring of the field. Furthermore, the diversity of indicator values represented by the species of arable weeds and wild plants suggest a low level of competition in the field, which is characteristics for ruderals thriving in highly disturbed habitats (Grime, 1977).

In summary, the reconstruction of cultivation practices and soil conditions of the material from Schwäbisch Hall indicates that the rye was sown as a winter cereal in highly disturbed fields with a moist, acidic to neutral soil that was likely manured. These conditions are similar to the ones reflected by the material from Göttingen, with the exception of the nitrogen content of the soil, which in the case of Göttingen did not suggest the practice of manuring.

References:

Bogaard, A.; Jones, G.; Charles, M. The impact of crop processing on the reconstruction of crop sowing time and cultivation intensity from archaeobotanical evidence. Veg. Hist. Archaeobot. 2005, 25, 505–509.

Charles, M.; Bogaard, A.; Jones, G.; Hodgson, J.; Halstead, P. Towards the archaeobotanical identification of intensive cereal cultivation: Present-day ecological investigation in the mountains of Asturias, northwest Spain. Veg. Hist. Archaeobot. 2002, 11, 113–142.

Ellenberg, H., 1988. Vegetation Ecology of Central Europe, 4th ed.; Cambridge University Press: Cambridge, UK.

Hillman, G.C. Reconstructing crop husbandry practices from charred remains of crops. In Farming Practice in British Prehistory; Mercer, R.J., Ed.; Edinburgh University Press: Edinburgh, UK, 1981; pp. 123–162.

Jones, G.; Charles, M.; Bogaard, A.; Hodgon, J.G.; Palmer, C. The functional ecology of present-day arable weed floras and its applicability for the identification of past crop husbandry. Veg. Hist. Archaeobot. 2005, 14, 493–504.

Klotz, S.; Kühn, I., Durka, W., 2002. BIOLFLOR—Eine Datenbank zu biologisch-ökologischen Merkmalen der Gefäßpflanzen in Deutschland. Schriftenreihe für Vegetationskunde 38, Bundesamt für Naturschutz: Bonn, Germany.

Kühn, I., Durka, W., Klotz, S., 2004. BiolFlor: a new plant-trait database as a tool for plant invasion ecology. Divers. Distrib. 10, 363–365.

Kreuz, A.; Schäfer, E.Weed finds as indicators for the cultivation regime of the early Neolithic Bandkeramik culture? Veg. Hist. Archaeobot. 2011, 20, 333–348.

Leuschner, C.; Ellenberg, H. Ecology of Central European Non-Forest Vegetation: Coastal to Alpine, Natural to Man-Made Habitats, Vegetation Ecology of Central Europe, 6th ed.; Springer: Cham, Germany, 2017; Volume 2.

Neveu, E.; Zech-Matterne, V.; Brun, C.; Dietsch-Sellami, M.; Durand, F.; Toulemonde, F. New insights into agriculture in northwestern France from the Bronze Age to the Late Iron Age: A weed ecological approach. Veg. Hist. Archaeobot. 2021, 30, 47–61.

Van Der Veen, M. Crop Husbandry Regimes: An Archaeobotanical Study of Farming in Northern England; Sheffield Archaeological Monographs: Sheffield, UK, 1992.
